# Qualitative Analysis of Novel Flavonoid Adducts from Nerve Agent Tabun-Exposed *Arabidopsis thaliana* (L.) Based on Quadrupole–Time of Flight Mass Spectrometry

**DOI:** 10.3390/molecules28062581

**Published:** 2023-03-12

**Authors:** Zhongfang Xing, Ruiqian Zhang, Zhehui Zhao, Ling Yuan, Huilan Yu, Yang Yang, Yuntao Yang, Shilei Liu, Chengxin Pei

**Affiliations:** 1 State Key Laboratory of NBC Protection for Civilian, Beijing 102205, China; 2 State Key Laboratory of Bioactive Substances and Functions of Natural Medicines, Department of Medicinal Chemistry, Institute of Materia Medica, Chinese Academy of Medical Sciences & Peking Union Medical College, Beijing 100050, China

**Keywords:** flavonoid, nerve agent, Tabun, *Arabidopsis thaliana* (L.), Q–TOF MS, plant biomarker

## Abstract

Flavonoids are a kind of secondary metabolite which widely exist in plants. They contain a lot of active hydroxyls, which can react with toxic chemicals to produce potential exposure biomarkers. In this article, the model plant *Arabidopsis thaliana* (L.) was exposed to the nerve agent O-Ethyl *N*,*N*-dimethyl phosphoramidocyanidate (Tabun). By comparing with the plant not exposed to Tabun, some characteristic ions were identified by quadrupole–time of flight mass spectrometry in the acetonitrile extract of the exposed leaves. These characteristic ions were selected as parent ions to produce product ion mass spectra (PIMS). Some interesting fragmentation pathways were revealed, including neutral loss of glucoside, rhamnose and ethylene. O-Ethyl *N*,*N*-dimethyl phosphoryl modified flavonoids were deduced from assignment of the PIMS. The element components and the accurate mass of the product ions from each parent ion matched well with those of the proposed fragmentation pathways. Through comparison with the PIMS of structurally closely related chemical of Isobutyl methylphosphonyl modified flavonoids, the structures and the fragmentation pathways of the O-Ethyl *N,N*-dimethyl phosphoryl modified flavonoids were finally confirmed. Successfully finding and identifying these three specific exposure biomarkers in plants provided a new strategy for the retrospective analysis of organophosphorus exposure and forensic analysis.

## 1. Introduction

O-Ethyl *N*,*N*-dimethyl phosphoramidocyanidate (Tabun) is a typical G-type organophosphorus nerve agent (OPNAs) which can cause muscle twitching, miosis, convulsions, seizures and finally death [1,2]. This chemical was first obtained during the development of organophosphorus pesticides in Germany before World War II (WWII) [2]. Industrial scale production was carried out during WWII and about 10,000 to 30,000 tons of Tabun were produced [2]. In the Iran–Iraq War, Tabun was employed by Iraq on a large scale for the first time and thousands of people were injured by the nerve agent during that time [2]. Although the signature of the “Convention on the Prohibition of the Development, Production, Stockpiling and Use of Chemical Weapons and their Destruction” (Chemical Weapons Convention, CWC) [3,4] in 1997 by 193 countries has prohibited the use of Tabun and other OPNAs, its illegal use in terrorist attacks and regional conflicts is still a potential threat in the world due to the ease and low cost of preparation. Successful detection of OPNAs can play a pivotal role in case of a terrorist attack with nerve agents.

**Scheme 1:**
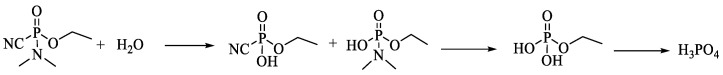
Hydrolysis pathway of Tabun.

Detection of Tabun generally relies upon environmental samples such as soil and water. Gas chromatography–mass spectrometry (GC–MS) is usually used to analyze the Tabun in environmental samples. However, in the environment, Tabun can react with moisture to yield the initial hydrolysis products ethyl *N*,*N*-dimethylphosphoramidic acid and ethyl phosphorocyanidic acid. These two initial hydrolysis products are unstable and will further degrade into ethyl phosphoric acid, which is unsuitable to be used as the indicator of Tabun because it is also a metabolite of some pesticides and plasticizers [5] (as shown in Scheme 1).

Biomedical samples are another type of samples for the verification of Tabun exposure. The most-used biomedical samples are urine and serum. In the urine, Tabun can react with water and undergo similar progress as in the environment (degrade into ethyl *N*,*N*-dimethylphosphoramidic acid, ethyl phosphorocyanidic acid and further into ethyl phosphoric acid), which makes the urine not suitable for retrospective detection. In the serum, Tabun can react with proteins (e.g., cholinesterase and albumin). For the adducted proteins, two analytical approaches are widely used. The first approach uses the fluoride ion to convert the phosphorylated moiety on the modified cholinesterase into O-Ethyl dimethylphosphoramidofluoridate [6,7,8], which are subsequently detected by GC–MS/MS. However, the phosphorylated moiety will be subject to hydrolysis or “aging” within hours to days, which makes regeneration impossible [5]. The second approach involves the isolation of adducted proteins in blood, followed by digestion and LC–MS/MS analysis of a specific adducted peptide, which contains the phosphorylated site [5,9]. However, some proteins (such as albumin) have a short half-life (for albumin, about 20 days), which makes it impossible for long-term retrospective detection [10].

Plants have probably become a new potential matrix used for the verification of chemical weapon exposure because of their wide existence in the environment. Toxic substances can cause morphological changes on plants, which makes plants potential sentinel species [11]. Because of their availability and no animal ethical issues, plants attracted the attention of the Organisation for the Prohibition of Chemical Weapons (OPCW) [11]. In recent years, OPCW has launched a challenge among laboratories worldwide to investigate new kinds of exposure biomarkers in plants. There are abundant components in plants, some of which may react with xenobiotic toxicants to produce characteristic exposure biomarkers [12]. However, reported researches mainly focused on the absorption and transformation of OPNA degradation products among different parts of plants [13,14,15,16].

Our previous research [17] proved that Isobutyl methylphosphonyl-modified flavonoids can be formed when the model plant *Arabidopsis thaliana* (L.) was exposed to a typical V-type nerve agent of Isobutyl S-2-diethylaminoethyl methylphosphonothiolate (iBuVX). The binding site of the iBuVX on flavonoids was proved to be the hydroxyl group on the benzene ring of the flavonoids by density functional theory computation and by the synthesis of the reference chemical. Because it is very difficult to synthesize and obtain every reference of the analytical target, especially the complicated adduct biomarkers in biomedical or plant matrices, comparison with the structurally closely related chemical has provided an alternative strategy to identify the new analytical target in the exposed specimen [18].

In this article, to the best of our knowledge, the potential biomarkers from the nerve agent of Tabun-exposed model plant *Arabidopsis thaliana* (L.) were investigated and three flavonoids modified by Tabun were identified for the first time. The three flavonoid adducts with O-Ethyl *N,N*-dimethyl phosphoryl moiety were detected and identified by liquid chromatography–Quadrupole–Time of Flight (LC–Q–TOF) mass spectrometry. The structures and the fragmentation pathways of the O-Ethyl *N*,*N*-dimethyl phosphoryl modified flavonoids were confirmed by comparison with the product ion mass spectra (PIMS) of the structurally closely related chemical O-Isobutyl methylphosphonyl-modified flavonoids. The analytical results are very important for the retrospective analysis of organophosphorus exposure, and it also provides a new model for the investigation of new potential biomarkers in plants exposed by xenobiotic toxicants.

## 2. Results and Discussion

### 2.1. Identification of Flavonoids in Arabidopsis thaliana (L.)

**Figure 1:**
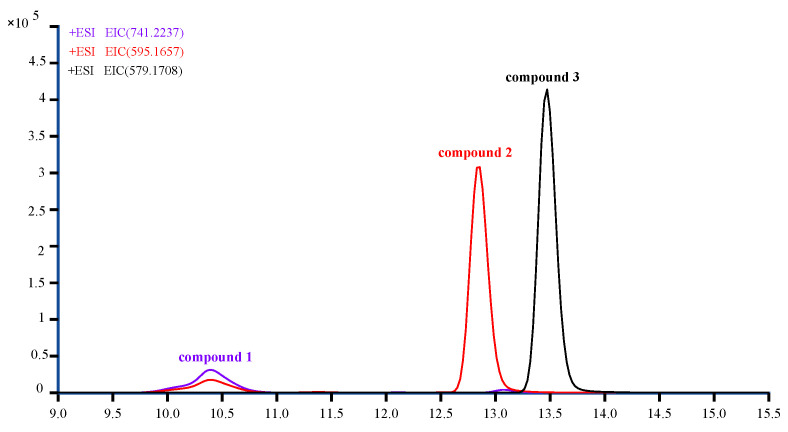
[M + H]^+^ EICs of three flavonoids identified in the *Arabidopsis thaliana* (L.) not exposed to Tabun.

**Figure 2:**
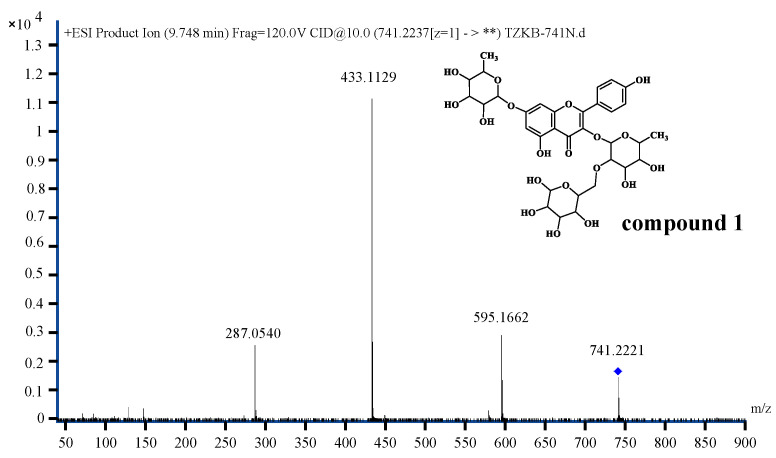
PIMS of kaempferol-3-O-rhamnosyl-glucoside-7-O-rhamnoside (compound **1**). The blue sign was used to mark the precursor ion.

**Figure 3:**
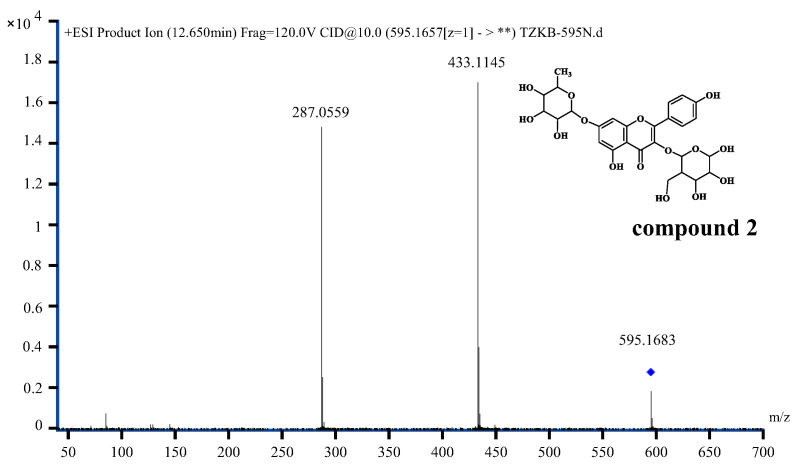
PIMS of kaempferol 3-O-glucoside 7-O-rhamnoside (compound **2**). The blue sign was used to mark the precursor ion.

**Figure 4:**
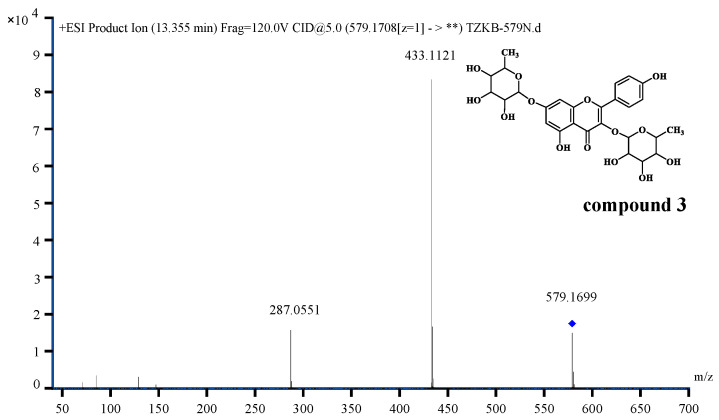
PIMS of kaempferol 3-O-rhamnoside 7-O-rhamnoside (compound **3**). The blue sign was used to mark the precursor ion.

Flavonoids are a kind of secondary metabolite of plants that widely exist in different plants [19,20]. They play important roles in plant growth, development, flowering, fruiting and disease prevention. Flavonoids contain a lot of active hydroxyls which may react with nerve agents to produce potential biomarkers. Q–TOF MS was used to analyze flavonoids in the acetonitrile extract of *Arabidopsis thaliana* (L.) leaves that were not exposed to Tabun. Both full scan and product ion scan modes were performed. The extracted ion chromatograms (EICs) of the quasi-molecular ions ([M + H]^+^) from three flavonoids not exposed to Tabun are shown in Figure 1. The [M + H]^+^ *m*/*z* value of compound **1**, compound **2** and compound **3** were calculated as 741.2237, 595.1657 and 579.1708, respectively. The collision energy was optimized to produce the PIMS with good quality. The corresponding PIMS are shown in Figure 2, Figure 3 and Figure 4, respectively.

For compound **1**, *m*/*z* 741.2237 was set as parent ion and three product ions were formed in the PIMS (Figure 2). The neutral loss between *m*/*z* 741 and *m*/*z* 595 was 146, which means compound **1** could have a rhamnose structure (rhamnose, C_6_H_12_O_4_, 148 Da). The neutral loss between *m*/*z* 741 and *m*/*z* 433 was 162 + 146, which means compound **1** could have a glucoside structure (glucoside, C_6_H_12_O_5_, 164 Da). The neutral loss between *m*/*z* 741 and *m*/*z* 287 was 162 + 146 + 146, which means compound **1** could have another rhamnose structure. The product ion *m*/*z* 287 was formed from the kaempferol structure (kaempferol, C_15_H_10_O_6_, 286 Da). The fragmentation patterns were consistent with references [21,22], and the substance was identified as kaempferol-3-*O*-rhamnosyl-glucoside-7-*O*-rhamnoside (C_33_H_40_O_19_). This chemical was reported to be present in *Arabidopsis thaliana* (L.) [21].

For compound **2** and compound **3**, similar neutral losses were observed in the PIMS. In the PIMS of compound **2** (Figure 3), the parent ion was set as *m*/*z* 595.1657 and two product ions were formed. The neutral loss between *m*/*z* 595 and *m*/*z* 433 was 162 and the neutral loss between *m*/*z* 595 and *m*/*z* 287 was 162 + 146, which means compound **2** could have a glucoside structure and a rhamnose structure. In the PIMS of compound **3** (Figure 4), the parent ion was set as *m*/*z* 579.1708 and two product ions were formed. The neutral loss between *m*/*z* 579 and *m*/*z* 433 was 146 and the neutral loss between *m*/*z* 579 and *m*/*z* 287 was 146 + 146, which means compound **3** could have two rhamnose structures. According to the fragmentation pathways, compound **2** was deduced to be kaempferol 3-*O*-glucoside 7-*O*-rhamnoside (C_27_H_30_O_15_) and compound **3** to be kaempferol 3-*O*-rhamnoside 7-*O*-rhamnoside (C_27_H_30_O_14_). These two chemicals were also reported to be present in *Arabidopsis thaliana* (L.) [21]. The deviations between the calculated *m*/*z* and observed *m*/*z* values of each fragment ions derived from compound **1**, compound **2** and compound **3** are in Table 1 (within 3.55 ppm), Table 2 (within 4.30 ppm) and Table 3 (within 1.91 ppm), respectively.

After the identification of the three flavonoids, comparison was made for the peak areas in EICs of the corresponding ([M + H]^+^) ions. The EICs are shown in Figure 1, in which the peak area of *m*/*z* 579.1708 (compound **3**) was the largest, while the peak area of *m*/*z* 741.2237 (compound **1**) was the least. Since the three flavonoids have similar chemical structures and similar properties, it can be inferred that among the three identified flavonoids, compound **3** was the most abundant in *Arabidopsis thaliana* (L), while compound **1** was the least.

### 2.2. Identification of Flavonoid Adducts Formed in Arabidopsis thaliana (L.) Exposed to Tabun

The discovery of long-term biomarkers is of great significance since it can provide convincible confirmation of nerve agent exposure. In this article, three flavonoid adducts as novel potential exposure biomarkers were identified in the extracts of leaves of *Arabidopsis thaliana* (L.) exposed to the nerve agent Tabun. These adducts can be detected four weeks after exposure, which makes them suitable for long-term retrospective detection.

**Figure 5:**
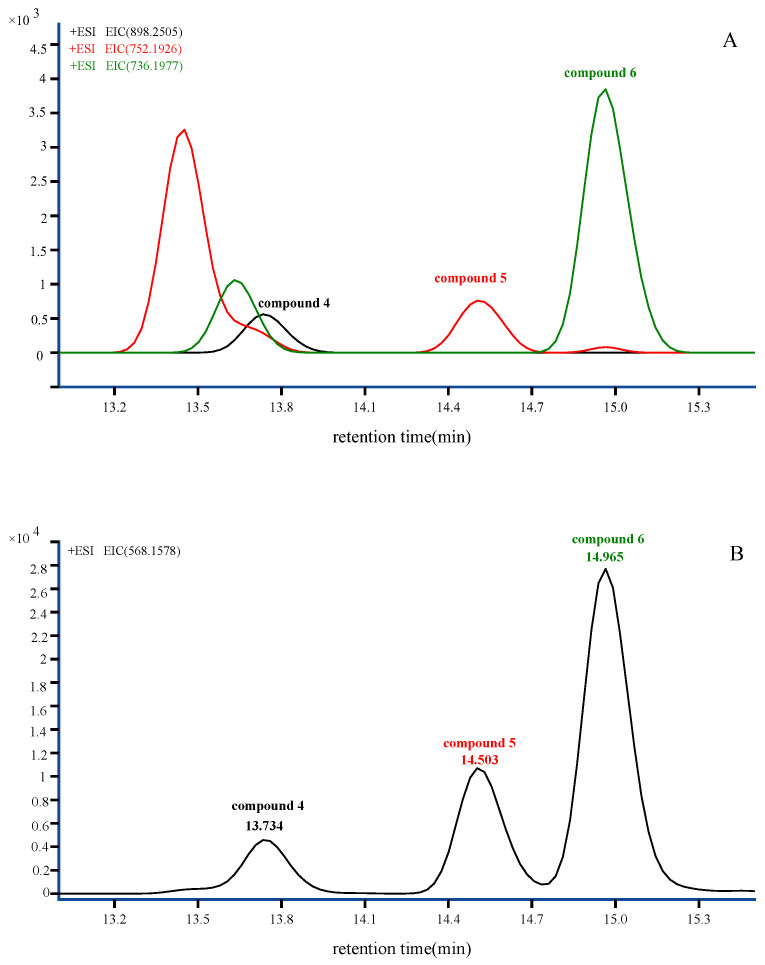
EICs of characteristic ions [M + Na]^+^ (**A**) and *m*/*z* 568.1578 (**B**) found in the extract of *Arabidopsis thaliana* (L.) exposed to Tabun.

In this article, Q–TOF MS was used to analyze the acetonitrile extract of both the exposed and unexposed *Arabidopsis thaliana* (L.) leaves. Both full scan and product ion scan modes were performed. By comparing the exposed plant with the plant not exposed to Tabun, two characteristic ions were identified in the full scan mass spectra at retention times of about 13.73 min, 14.50 min and 14.96 min, respectively. Figure 5A shows EICs of the quasi-molecular ion ([M + Na]^+^) of the three flavonoid adducts, which were coded as compound four, compound five and compound six, of which the *m*/*z* values were 898.2505, 752.1926 and 736.1977, respectively. Besides the quasi-molecular ion ([M + Na]^+^) of the three flavonoid adducts, the characteristic ion *m*/*z* 568.1578 (EICs shown in Figure 5B) was also found in the corresponding retention times of the three compounds.

**Figure 6:**
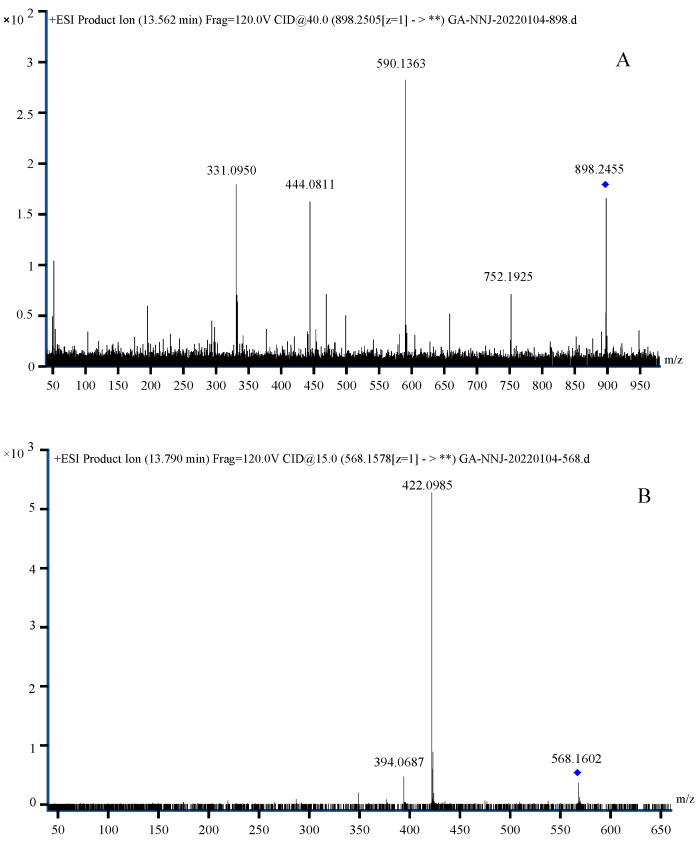
PIMS of *m*/*z* 898.2505 (**A**) and *m*/*z* 568.1578 (**B**) found in the extract of *Arabidopsis thaliana* (L.) exposed to Tabun. The blue sign was used to mark the precursor ion.

**Figure 7:**
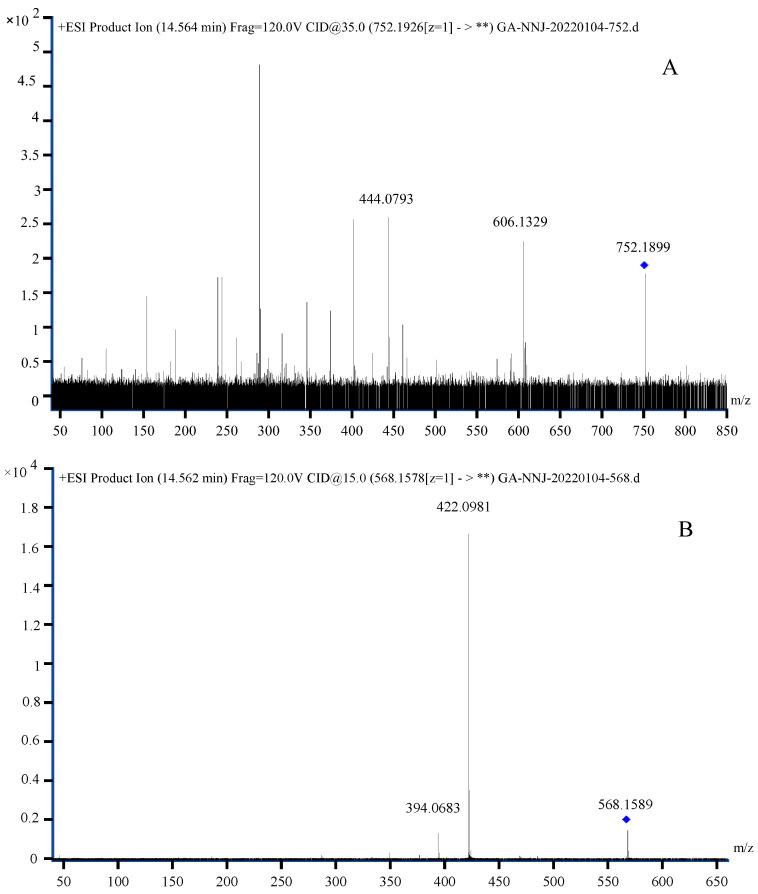
PIMS of *m*/*z* 752.1926 (**A**) and *m*/*z* 568.1578 (**B**) found in the extract of *Arabidopsis thaliana* (L.) exposed to Tabun. The blue sign was used to mark the precursor ion.

**Figure 8:**
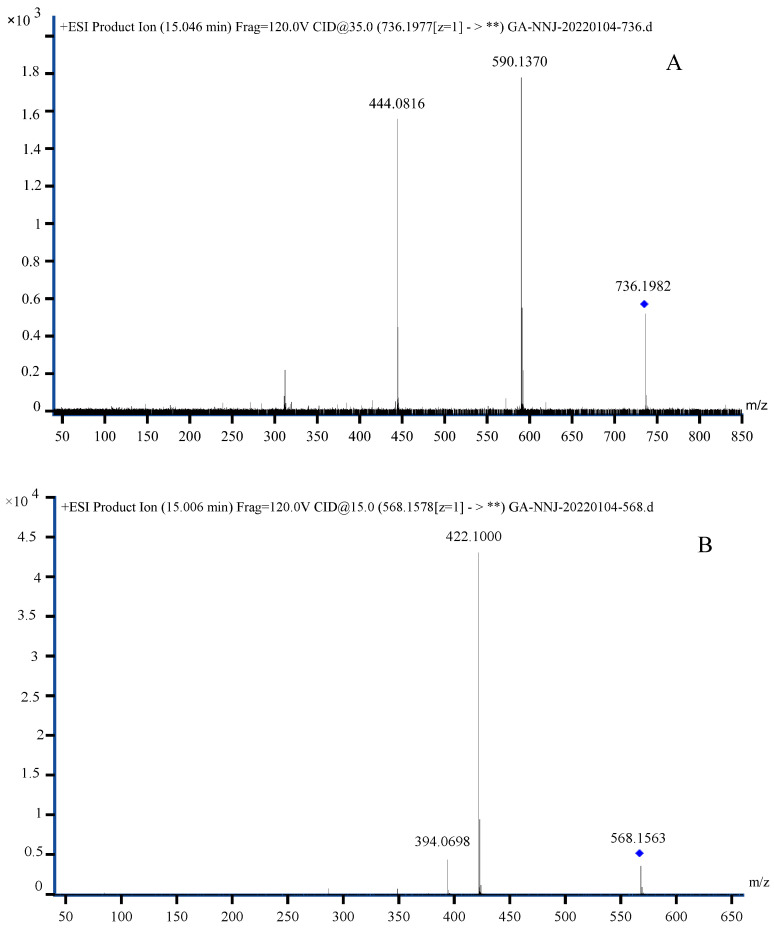
PIMS of *m*/*z* 736.1977 (**A**) and *m*/*z* 568.1578 (**B**) found in the extract of *Arabidopsis thaliana* (L.) exposed to Tabun. The blue sign was used to mark the precursor ion.

Compound four, compound five and compound six were the reaction products of Tabun with compound **1**, compound **2** and compound **3**, respectively. There were nine hydroxyls located on the sugar rings and two located on the benzene rings. In order to specify the reaction sites, PIMS of the quasi-molecular ion ([M + Na]^+^) and characteristic ion *m*/*z* 568.1578 were collected with collision energy optimized. For compound four, compound five and compound six, the PIMS are shown in Figure 6, Figure 7 and Figure 8.

In Figure 6A, the parent ion was set as m/z 898.2505 and four product ions were formed. The parent ion *m*/*z* 898 lost 146, leading to the formation of *m*/*z* 752; lost 146 + 162, leading to the formation of *m*/*z* 590; and lost 146 + 146 + 162, leading to the formation of *m*/*z* 444. The product ion *m*/*z* 331 was formed from the cationized disaccharide fragment. In Figure 7A, the parent ion was set as *m*/*z* 752.1926 and two product ions were formed. The parent ion *m*/*z* 752 lost 146, leading to the formation of *m*/*z* 606, and lost 146 + 162, leading to the formation of *m*/*z* 444. In Figure 8A, the parent ion was set as *m*/*z* 736.1977 and two product ions were formed. The parent ion *m*/*z* 736 lost 146 leading to the formation of *m*/*z* 590, and lost 146 + 146, leading to the formation of *m*/*z* 444. In Figure 6B, Figure 7B and Figure 8B, the parent ion was set as *m*/*z* 568.1578 and two product ions were formed. The parent ion *m*/*z* 568 lost 146, leading to the formation of *m*/*z* 422, and lost 146 + 28, leading to the formation of *m*/*z* 394. The neutral losses 146, 162 and 28 were due to the departure of rhamnose, glucoside and ethylene moieties through the respective fragmentations. From the fragmentation behaviors, it can be deduced that the modified site was located on a benzene ring instead of a sugar ring.

**Figure 9:**
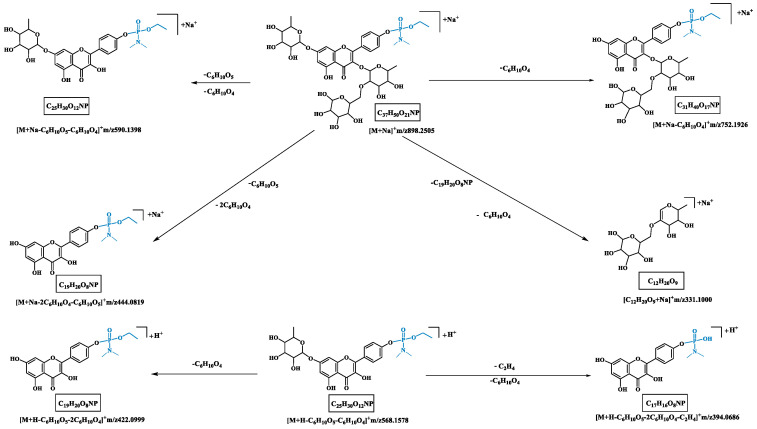
Possible fragmentation pathways of product ions for compound **4**.

**Figure 10:**
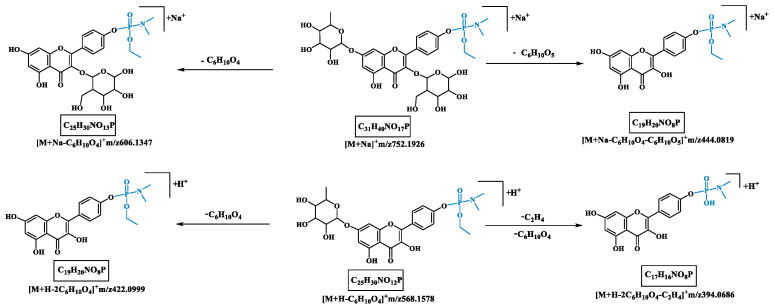
Possible fragmentation pathways of product ions for compound **5**.

**Figure 11:**
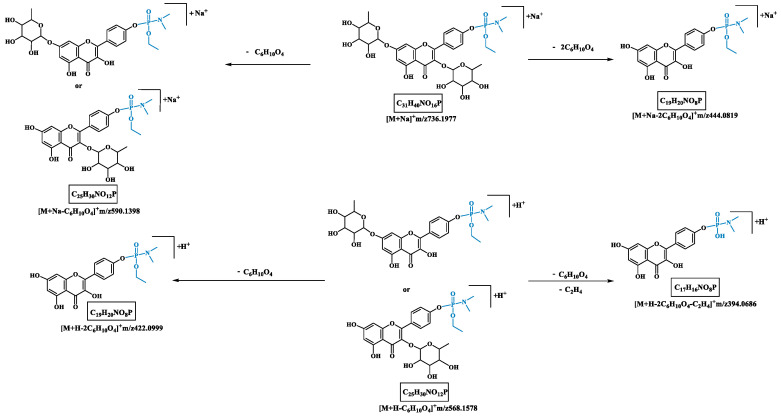
Possible fragmentation pathways of product ions for compound **6**.

In our previous research [17], density functional theory computation was used to compare the reactivity of the two hydroxyl groups on the benzene ring of compound **1**, compound **2** and compound **3**. It was found that O18 has relatively low steric hindrance and stronger nucleophilicity than O20, and is more likely to attack the P atom in the molecule of a nerve agent. Possible structures (O18 being the modified site) of the O-Ethyl *N,N*-dimethyl phosphoryl modified flavonoids and fragmentation pathways of product ions are illustrated in Figure 9, Figure 10 and Figure 11, respectively. The deviations between the calculated and observed *m*/*z* values of each fragment derived from compound four, compound five and compound six are shown in Table 4 (within 6.14 ppm), Table 5 (within 6.11 ppm) and Table 6 (within 4.91 ppm), respectively.

### 2.3. Confirmation of the Identification of Flavonoid Adducts by a Structurally Closely Reference

**Figure 12:**
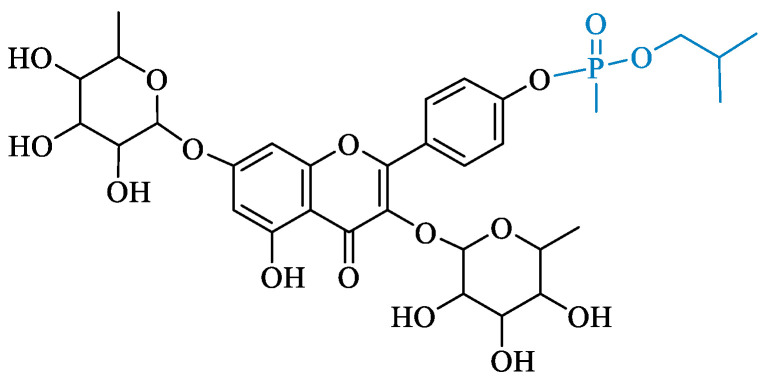
Structure of the synthesized of Isobutyl methylphosphonyl-modified flavonoid (compound **7**).

**Figure 13:**
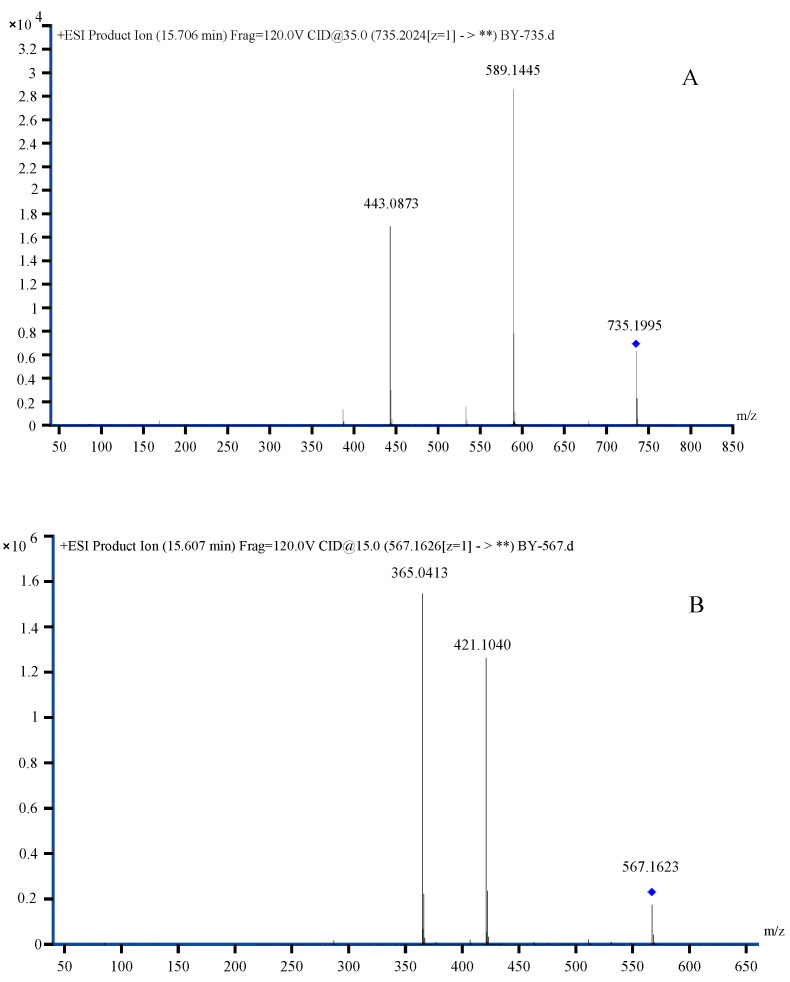
PIMS of *m*/*z* 735.2024 (**A**) and *m*/*z* 567.1626 (**B**) from the reference of compound **7**. The blue sign was used to mark the precursor ion.

Identification of chemicals by references is reliable and is usually used by chemists. In the field of verification for chemical weapon abuse, the identification of unknown chemicals is usually based on comparison with authentic reference chemicals or recorded spectra in the database [18,23]. In our previous work, we identified the new phosphonyl-modified flavonoid adducts by comparison with the synthesized reference, in which the synthetic route was complicated and laborious. It is not easy to synthesize and obtain every reference chemical, especially for the complicated adduct biomarkers of chemical agents with lethal toxicity. Therefore, the OPCW provided a third approach to identify the unknown chemicals by comparison with structurally closely related chemicals. When used for comparison, the spectra of the structurally closely related chemicals together with spectral interpretations must be provided [18]. In order to confirm the identification of the structures of the three adducts without references chemicals, comparison was made to a structurally closely related reference of Isobutyl methylphosphonyl-modified flavonoid (compound seven, the structure is shown in Figure 12), which was synthesized in-house previously. The PIMS of [M + Na]^+^ *m*/*z* 735.2024 and [M + H − C_6_H_10_O_4_]^+^ *m*/*z* 567.1626 of compound seven was collected as shown in Figure 13. The deviations between the calculated and observed *m*/*z* values of each fragment were within 4.13 ppm, as shown in Table 7.

**Figure 14:**
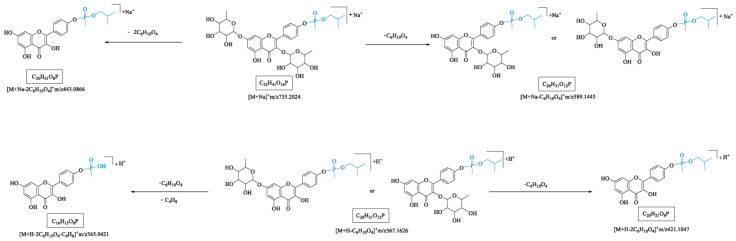
Possible fragmentation pathways of product ions for compound **7**.

In Figure 13A, the parent ion was set as *m*/*z* 735.2024 and two product ions were formed. The parent ion *m*/*z* 735 lost 146, leading to the formation of *m*/*z* 589, and lost 146 + 146, leading to the formation of *m*/*z* 443. In Figure 13B, the parent ion was set as *m*/*z* 567.1626 and two product ions were formed. The parent ion *m*/*z* 567 lost 146, leading to the formation of *m*/*z* 421, and lost 146 + 56, leading to the formation of *m*/*z* 365. The neutral losses 146 and 56 were due to the departure of rhamnose and butylene moieties through the respective fragmentations. The possible fragmentation pathways of compound seven are shown in Figure 14.

By comparing the PIMS and fragmentation pathways of compound four (Figure 6 and Figure 9), compound five (Figure 7 and Figure 10), compound six (Figure 8 and Figure 11) and compound seven (Figure 13 and Figure 14), we found that the four chemicals were similar in that there were fragments resulting from the neutral loss of saccharide and alkene structural units. This result showed that compound four, compound five, compound six and compound seven have similar structures, thus confirming the chemical structures of the O-Ethyl *N,N*-dimethyl phosphoryl modified flavonoids.

After identifying the three new adducts, the peak area of the three adducts were compared. For adducts compound four, compound five and compound six, they are the reaction products of Tabun with compound **1**, compound **2** and compound **3**, respectively. Figure 5 shows that the peak area of compound six (Tabun-modified compound **3**) was the highest, while the peak area of compound four (Tabun-modified compound **1**) was the least, which is consistent with that of the three flavonoids. The results indicated that among the endogenous components, which have similar chemical structures and similar properties, the xenobiotic toxicants tend to react with the abundant endogenous components and form the exposure biomarkers. Therefore, among the endogenous components that have similar chemical structures and similar properties that can react with xenobiotic toxicants, more attention should be paid to the abundant endogenous components.

## 3. Experiment

### 3.1. Reagents and Materials

Kaempferol 3-*O*-rhamnoside 7-*O*-rhamnoside (99.5%) was purchased from Shanghai Yuanye Bio-Technology, Co., Ltd. (Shanghai, China), and was used as received. All solvents were purchased from Sigma Aldrich, Co., Ltd. (Milwaukee, WI, USA) either in HPLC–MS grade or in analytical grade. Tabun was provided by the Laboratory of Analytical Chemistry, Research Institute of Chemical Defence. Isobutyl methylphosphonyl-modified flavonoids were synthesized with kaempferol 3-*O*-rhamnoside 7-*O*-rhamnoside and Isobutyl methylphosphonochloridate. The structures and purities (>95%) were confirmed by NMR [17].

### 3.2. Plant Culture and Nerve Agent Exposure

*Arabidopsis thaliana* (L.) (ecotype Columbia) was grown in a controlled greenhouse environment with an average temperature of 25 °C, 65% relative humidity and 9 h of light (100 μmol·m^−2^·s^−1^) every 24 h. Tabun (about 1–2 mg) was administrated to the leaves of the five-week-old plants, which were cultured for seven more days.

### 3.3. Preparation of Plant Extracts

The leaves from exposed *Arabidopsis thaliana* (L.) (about 20–30 mg) were harvested and cut into small pieces into a 5 mL centrifugation tube. For the extraction, 1.0 mL of acetonitrile was added to the plant materials and the sample was homogenized using a high-speed disperser (Type XHF-DY, NINGBO Scientz Bitotechnology, Co., LTD, Ningbo, China) for 1 min at 5000 r/min. Afterwards, the samples were vortexed for 10 min and centrifuged for 5 min at 13,500 r/min. Supernatants were separated from the solid residues and kept at −20 °C until analysis with LC–Q–TOF mass spectrometry.

### 3.4. LC–Q–TOF MS Analyses

LC–Q–TOF MS analyses were performed on a 1200 HPLC system coupled to an Agilent 6520 Q–TOF mass spectrometer (Agilent Technologies, Santa Clara, CA, USA). A Zorbax Eclipse Plus C18 column with dimensions of 150 mm × 2.1 mm and 5.0 μm particle size was used at 30 °C. A gradient elution was applied using 0.1% formic acid solution in water as solvent (A) and 0.1% formic acid solution in acetonitrile as solvent (B). The initial condition was set at 2% of B. The following solvent gradient was applied: from 2% B to 12% B within 2 min, to 15% B within 6 min, to 60% B within 9 min, to 100% B within 9 min, to 2% B within 1 min, and hold for 5 min. The flow rate was set at 0.25 mL/min and 1–5 μL of samples was injected into the instrument using an autosampler. Mass resolution was set to 10,000. The electrospray and fragmentor voltages were set at 3500 V and 120 V, respectively. The gas temperature was maintained at 350 °C. The drying gas (nitrogen) flow rate and nebulizer gas (nitrogen) pressure were 8 L/min and 30 psi, respectively. MS/MS product ion scans were carried out at a collision energy of 5–45 V. Ultra-high-purity nitrogen was used as collision gas. The scan was performed in positive mode in the *m*/*z* range 25–1000 with a scan time of 0.77 s in centroid mode.

### 3.5. Safety Consideration

As a highly lethal chemical, Tabun should be handled carefully. The preparation of Tabun solutions and the exposed *Arabidopsis thaliana* (L.) should be performed in a fume hood by professionals with appropriate protective equipment. All materials in direct contact with Tabun should be decontaminated thoroughly with a bleach solution.

There is no risk in preparing and analyzing the modified flavonoids. The plant extracts should be treated following universal safety precautions for handling environmental samples.

## 4. Conclusions

Three novel flavonoid adducts in Tabun-exposed *Arabidopsis thaliana* (L.) were identified based on the full scan and PIMS of Q–TOF MS. The structures of the O-Ethyl *N,N*-dimethyl phosphoryl modified flavonoids and the fragmentation pathways were proposed and discussed. The calculated *m*/*z* from the proposed fragmentation pathways and observed *m*/*z* from the product ions of each parent ion matched well. The structures of the O-Ethyl *N,N*-dimethyl phosphoryl modified flavonoid adducts were confirmed by interpretation of the PIMS of a structurally closely related chemical of Isobutyl methylphosphonyl modified flavonoid, which has been identified as a plant exposure biomarker of iBuVX. This strategy of using structurally closely related chemical is recommended by OPCW and is often used in the fields of chemical weapons verification. However, identification of chemicals only by reference spectra has a different level of reliability; it can sometimes lead to false identification. In order to improve the identification reliability, different biomarkers and more techniques are needed.

Successful screening and identification of the specific exposure biomarkers of flavonoid adducts makes it possible for the retrospective analysis of toxic chemical exposure through the plant matrices. The finding of exposure biomarkers in plants provided a new strategy for the retrospective analysis of nerve agent exposure. However, different nerve agents have different reactivities when reacting with plant components, and there are many other endogenous plant components that can react with nerve agents, so more research on different OPNAs and different plants is needed in future work. Meanwhile, the identification of chemicals by structurally closely related chemicals in this work provided an optional strategy for chemical identification when neither reference chemicals nor reference spectra are available. This optional strategy may be shared by scientists in other fields associated with chemical identification in addition to the verification of chemical weapon conventions.

## Figures and Tables

**Table 1:** The deviations between calculated and observed *m*/*z* values of each fragment derived from compound **1**.

Fragment	Calculated *m*/*z*	Observed *m*/*z*	Deviation
[M + H]^+^	741.2237	741.2221	−2.10 ppm
[M + H − C_6_H_10_O_4_]^+^	595.1657	595.1662	0.76 ppm
[M + H − C_6_H_10_O_4_ − C_6_H_10_O_5_]^+^	433.1129	433.1129	−0.05 ppm
[M + H − 2C_6_H_10_O_4_ − C_6_H_10_O_5_]^+^	287.0550	287.0540	−3.55 ppm

**Table 2:** The deviations between calculated and observed *m*/*z* values of each fragment derived from compound **2**.

Fragment	Calculated *m*/*z*	Observed *m*/*z*	Deviation
[M + H]^+^	595.1657	595.1683	4.30 ppm
[M + H − C_6_H_10_O_4_]^+^	433.1129	433.1145	3.65 ppm
[M + H − C_6_H_10_O_4_ − C_6_H_10_O_5_]^+^	287.0550	287.0559	3.10 ppm

**Table 3:** The deviations between calculated and observed *m*/*z* values of each fragment derived from compound **3**.

Fragment	Calculated *m*/*z*	Observed *m*/*z*	Deviation
[M + H]^+^	579.1708	579.1699	−1.61 ppm
[M + H − C_6_H_10_O_4_]^+^	433.1129	433.1121	−1.91 ppm
[M + H − 2C_6_H_10_O_4_]^+^	287.0550	287.0551	0.30 ppm

**Table 4:** The deviations between calculated and observed *m*/*z* values of each fragment derived from compound **4**.

Fragment	Calculated *m*/*z*	Observed *m*/*z*	Deviation
[M + Na]^+^	898.2505	898.2455	−5.73 ppm
[M + Na − C_6_H_10_O_4_]^+^	752.1926	752.1925	−0.15 ppm
[M + Na − C_6_H_10_O_4_ − C_6_H_10_O_5_]^+^	590.1398	590.1363	−6.14 ppm
[M + H − C_6_H_10_O_4_ − C_6_H_10_O_5_]^+^	568.1578	568.1602	4.16 ppm
[M + Na − 2C_6_H_10_O_4_ − C_6_H_10_O_5_]^+^	444.0819	444.0811	−1.84 ppm
[M + H − 2C_6_H_10_O_4_ − C_6_H_10_O_5_]^+^	422.0999	422.0985	−3.39 ppm
[M + H − 2C_6_H_10_O_4_ − C_6_H_10_O_5_ − C_2_H_4_]^+^	394.0686	394.0687	0.18 ppm

**Table 5:** The deviations between calculated and observed *m*/*z* values of each fragment derived from compound **5**.

Fragment	Calculated *m*/*z*	Observed *m*/*z*	Deviation
[M + Na]^+^	752.1926	752.1899	−3.71 ppm
[M + Na − C_6_H_10_O_4_]^+^	606.1347	606.1329	−3.08 ppm
[M + H − C_6_H_10_O_5_]^+^	568.1578	568.1589	1.87 ppm
[M + Na − C_6_H_10_O_4_ − C_6_H_10_O_5_]^+^	444.0819	444.0793	−6.11 ppm
[M + H − C_6_H_10_O_4_ − C_6_H_10_O_5_]^+^	422.0999	422.0981	−4.34 ppm
[M + H − C_6_H_10_O_4_ − C_6_H_10_O_5_ − C_2_H_4_]^+^	394.0686	394.0683	−0.84 ppm

**Table 6:** The deviations between calculated and observed *m*/*z* values of each fragment derived from compound **6**.

Fragment	Calculated *m*/*z*	Observed *m*/*z*	Deviation
[M + Na]^+^	736.1977	736.1982	0.71 ppm
[M + Na − C_6_H_10_O_4_]^+^	590.1398	590.1370	−4.91 ppm
[M + H − C_6_H_10_O_4_]^+^	568.1578	568.1563	−2.71 ppm
[M + Na − 2C_6_H_10_O_4_]^+^	444.0819	444.0816	−0.65 ppm
[M + H − 2C_6_H_10_O_4_]^+^	422.0999	422.1000	0.17 ppm
[M + H − 2C_6_H_10_O_4_ − C_2_H_4_]^+^	394.0686	394.0698	2.98 ppm

**Table 7:** The deviations between calculated and observed *m*/*z* values of each fragment derived from compound **7**.

Fragment	Calculated *m*/*z*	Observed *m*/*z*	Deviation
[M + Na]^+^	735.2024	735.1995	−4.13 ppm
[M + Na − C_6_H_10_O_4_]^+^	589.1445	589.1445	−0.06 ppm
[M + H − C_6_H_10_O_4_]^+^	567.1626	567.1623	−0.51 ppm
[M + Na − 2C_6_H_10_O_4_]^+^	443.0866	443.0873	−1.61 ppm
[M + H − 2C_6_H_10_O_4_]^+^	421.1047	421.1040	−1.62 ppm
[M + H − 2C_6_H_10_O_4_ − C_4_H_8_]^+^	365.0421	365.0413	−2.14 ppm

## Data Availability

Not applicable.

## Sample Availability

Samples of the compounds are available from the authors.
